# CUMULATIVE STRESS EXPOSURE, NEIGHBORHOOD ENVIRONMENTS, AND INFLAMMATION IN ADULTHOOD

**DOI:** 10.1093/geroni/igae098.1223

**Published:** 2024-12-31

**Authors:** Jean Choi, Mateo Farina, Elizabeth Munoz

**Affiliations:** The University of Texas Austin, Austin, Texas, United States; University of Texas at Austin, Austin, Texas, United States; The University of Texas Austin, Austin, Texas, United States

## Abstract

Cumulative stress exposure, or the total stress exposure occurring across childhood and adulthood, is related to elevated inflammation, a key factor underlying physiological aging. Neighborhood contexts represent important sources of risk or resilience that may interact with stress to influence inflammation. Using data from the Health and Retirement Study, we assessed whether cumulative stress exposure would be associated with greater inflammation, and if this link is shaped by adult neighborhood cohesion and disorder (n = 5,625; age: 56 – 107). We constructed a continuous latent variable of systemic inflammation from six measured indicators (CRP, IL6, IL10, IL-1Ra, sTNFR1, IGF-1). Higher cumulative stress exposure was associated with elevated inflammation (b = 0.02, SE = 0.01, p =.032), but neighborhood contexts did not moderate this link. Sensitivity analyses revealed a significant interaction effect whereby greater neighborhood cohesion buffered the negative effect of cumulative stress exposure on IL-1Ra (b = -8.01, SE = 2.76, p =.004), and IGF-1 (b = -0.49, SE = 0.20, p =.014). Additionally, higher cumulative stress exposure was associated with elevated IL-1Ra in the context of greater neighborhood disorder (b = 4.43, SE = 2.24, p =.047). Results indicate that neighborhood cohesion and disorder are integral dimensions of the neighborhood environment that may modify the impact of cumulative stress exposure on markers of inflammation. These findings emphasize the need for additional research to consider an integrated multi-level approach to understand the effects of cumulative stress on inflammation.

